# Relief of endoplasmic reticulum stress enhances DNA damage repair and improves development of pre-implantation embryos

**DOI:** 10.1371/journal.pone.0187717

**Published:** 2017-11-03

**Authors:** Naomi Dicks, Rodrigo C. Bohrer, Karina Gutierrez, Marek Michalak, Luis B. Agellon, Vilceu Bordignon

**Affiliations:** 1 Department of Animal Science, McGill University, Ste-Anne-de-Bellevue, Quebec, Canada; 2 Department of Biochemistry, University of Alberta, Edmonton, Alberta, Canada; 3 School of Human Nutrition, McGill University, Ste-Anne-de-Bellevue, Quebec, Canada; University of Florida, UNITED STATES

## Abstract

Early-cleaving embryos are known to have better capacity to reach the blastocyst stage and produce better quality embryos compared to late-cleaving embryos. To investigate the significance of endoplasmic reticulum (ER) stress on early embryo cleavage kinetics and development, porcine embryos produced *in vitro* were separated into early- and late-cleaving groups and then cultured in the absence or presence of the ER stress inhibitor tauroursodeoxycholic acid (TUDCA). Developing embryos were collected at days 3 to 7 of culture for assessment of ER stress status, incidence of DNA double-strand breaks (DSBs), development and total cell number. In the absence of TUDCA treatment, late-cleaving embryos exhibited ER stress, higher incidence of DNA DSBs, as well as reductions in development to the blastocyst stage and total embryo cell numbers. Treatment of late-cleaving embryos with TUDCA mitigated these effects and markedly improved embryo quality and development. These results demonstrate the importance of stress coping responses in early developing embryos, and that reduction of ER stress is a potential means to improve embryo quality and developmental competence.

## Introduction

Embryo death during early developmental stages is a leading component of infertility. In livestock, most naturally fertilized oocytes are unable to survive beyond the implantation stage[1]. It is also well documented that less than 50% of embryos produced by *in vitro* fertilization (IVF) are able to establish pregnancy either in humans or livestock[2–4]. Such failures are attributable to many factors including gamete integrity and quality, genetic and genomic defects, and environmental factors affecting the embryo developmental milieu, such as the health, and hormonal and metabolic status of the zygote/embryo host[5–7].

During *in vitro* embryo production, distinct populations of embryos can be differentiated according to cleavage kinetics, which can predict developmental potential. Early-cleaving embryos begin to divide shortly after fertilization or activation, generally within the first 24 h in the porcine species, whereas late-cleaving embryos initiate their first cell division after 24 h[8, 9]. It has been observed in pigs and cattle that early-cleaving embryos have a greater ability to develop to the blastocyst stage and also have better quality, as assessed by total embryo cell numbers[9–13]. This improved developmental capacity has been shown in many mammalian species, including mice, pigs, cattle, hamsters and even humans[9–16]. In livestock, it is possible to accurately predict successful development to the blastocyst stage by evaluating embryo cell cleavage kinetics[12]. In humans, it has been shown that transfer of early-cleaving embryos produce higher pregnancy rates[16].

While it is now clear that cleavage kinetics represents an important parameter of embryo competence, the nature of these differences is not understood. Recently, Bohrer and colleagues showed that late-cleaving porcine embryos have increased DNA damage and this affects their quality and ability to reach the blastocyst stage[17]. DNA damage is an inciting cause of ER stress, a state of cellular dysfunction that is associated with aberration of multiple cellular metabolic pathways[18, 19]. Cells normally mount stress coping strategies, such as the unfolded protein response[20, 21] and genome damage response pathways[19, 22, 23], to regain homeostasis.

The ability to maintain cellular homeostasis throughout the execution of the metabolically active developmental program is paramount for ensuring the survival of the embryo. ER stress is of interest in development since important regulators of the ER stress response have been implicated in murine embryo survival or death[24–28]. Glucose-regulated protein 78kDa (GRP78) is known as the “master regulator” of the unfolded protein response given its ability to modulate the activation of the three main pathways involved in this coping mechanism[18, 20]. GRP78 has been shown to be essential for early embryo cell growth in mice[24]. One of the main arms activated by GRP78 is the inositol-requiring enzyme 1 (IRE1) pathway[18, 20]. This pathway has equally been implicated in murine embryo survival[28]. Dimerization and phosphorylation of IRE1 stimulates its endoribonuclease activity, which removes a 26-nucleotide fragment from the X-box binding protein 1 (XBP1) mRNA[18, 20]. This spliced form of XBP1 mRNA encodes the XBP1s form of the transcription factor that translocates to the nucleus to induce the expression of chaperones and other proteins important in the mitigation of ER stress[18, 20]. Functional XBP1s has been identified in murine and porcine developing embryos[29, 30], indicating inherent ER stress during preimplantation development in these species. Interestingly, treatment with the ER stress inhibitor, TUDCA, improves development in these models[29, 30]. There is also evidence that XBP1s regulates genes involved in the genome damage response[31, 32] and its knockdown results in increased foci of DNA damage[32].

While regulators of ER stress have been shown to be involved in development[24–28], to date, there has been no investigation into the impact of ER stress on embryo cleavage kinetics, an important indicator of embryo competence. It has been clearly shown in mice and pigs that induction of ER stress in embryos impedes their development [29, 30, 33], however, inherent ER stress status has not been evaluated in early- and late-cleaving embryos. Further insight into the nature of late-cleaving embryos can shed light on the cause of their poor development, as well as provide a means to improve fertility. The objective of the present study was to assess inherent ER stress status and DNA damage in both early- and late-cleaving porcine embryos, and determine if these factors affect embryo developmental capacity and quality.

## Results

**Fig 1:**
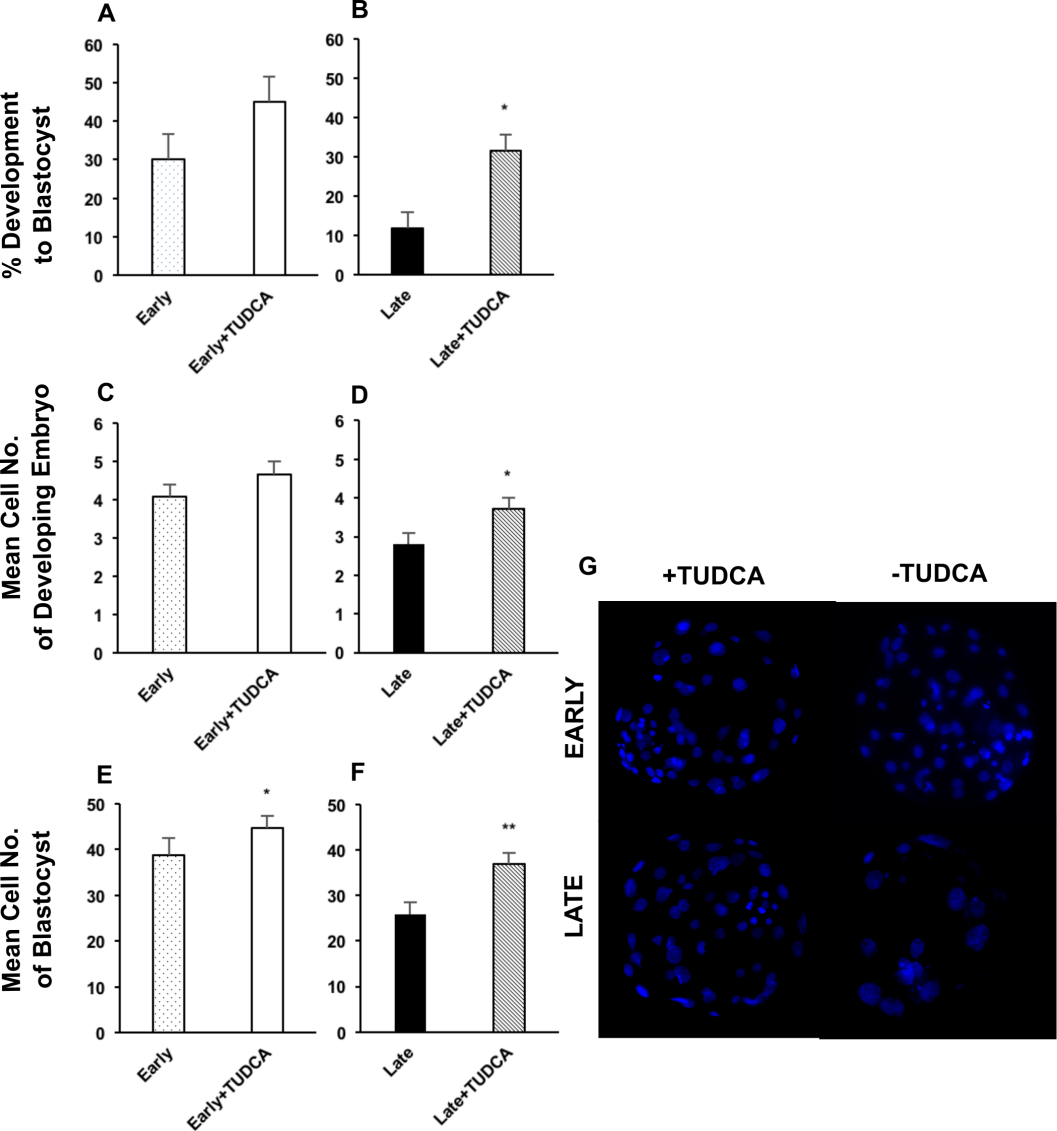
Culture with TUDCA improves embryo quality and blastocyst rate. Evaluation of embryo quality, as indicated by mean total cell number, was performed in developing embryos and blastocysts cultured without or with TUDCA. Development to the blastocyst stage was also investigated. Percent development to the blastocyst stage (blastocyst rate) in each treatment group of early-cleaving embryos, n = 335 (A) and late-cleaving embryos, n = 585 (B). Mean total cell number for each treatment group of early-cleaving developing embryos, n = 42 (C) and late-cleaving developing embryos, n = 42 (D). Mean total cell number for each treatment group of early-cleaving blastocysts, n = 89 (E) and late-cleaving blastocysts, n = 65 (F). Representative images of early-cleaving and late-cleaving blastocysts cultured without or with TUDCA (G). Images were captured at 20X magnification. Developing embryos are defined as those collected at day 3 and 5 of culture. Data was collected from 5 replicates for blastocyst development and 7 replicates for blastocyst cell number. Late = late-cleaving embryos cultured without TUDCA; Late + TUDCA = late-cleaving embryos cultured with TUDCA; Early = early-cleaving embryos cultured without TUDCA; Early + TUDCA = early-cleaving embryos cultured with TUDCA. Statistically significant differences among groups are indicated by * (p < 0.05) and ** (p < 0.01).

To assess the effects of resolving inherent ER stress on embryo development, both early- and late-cleaving embryos were treated with TUDCA, an ER stress inhibitor. Treatment of early-cleaving embryos with TUDCA did not result in a significant increase in the rate of development to the blastocyst stage (Fig 1A). However, the blastocyst rate of late-cleaving embryos increased 2.5 fold from 11.7% to 31.4% (Fig 1B). Interestingly, inhibition of ER stress increased development of late-cleaving embryos (31.4%) to a rate similar to untreated early-cleaving embryos (30.2%). These TUDCA-treated late-cleaving embryos, which normally develop poorly, had improved quality during both early development (Fig 1D) and at the blastocyst stage (Fig 1F and 1G), as indicated by an increase in mean total cell number by 32.1% (2.8 to 3.7, p = 0.02) and 43.0% (25.8 to 36.9, p = 0.004), respectively. Treatment of early-cleaving embryos did not improve quality in early development (Fig 1C), but did so at the blastocyst stage (Fig 1E and 1G). Importantly, TUDCA treatment increased the number of cells in late-cleaving embryos to similar numbers of untreated early-cleaving embryos at both the developing (3.7 vs. 4.1) and blastocyst stages (36.9 vs. 38.7), respectively.

**Fig 2:**
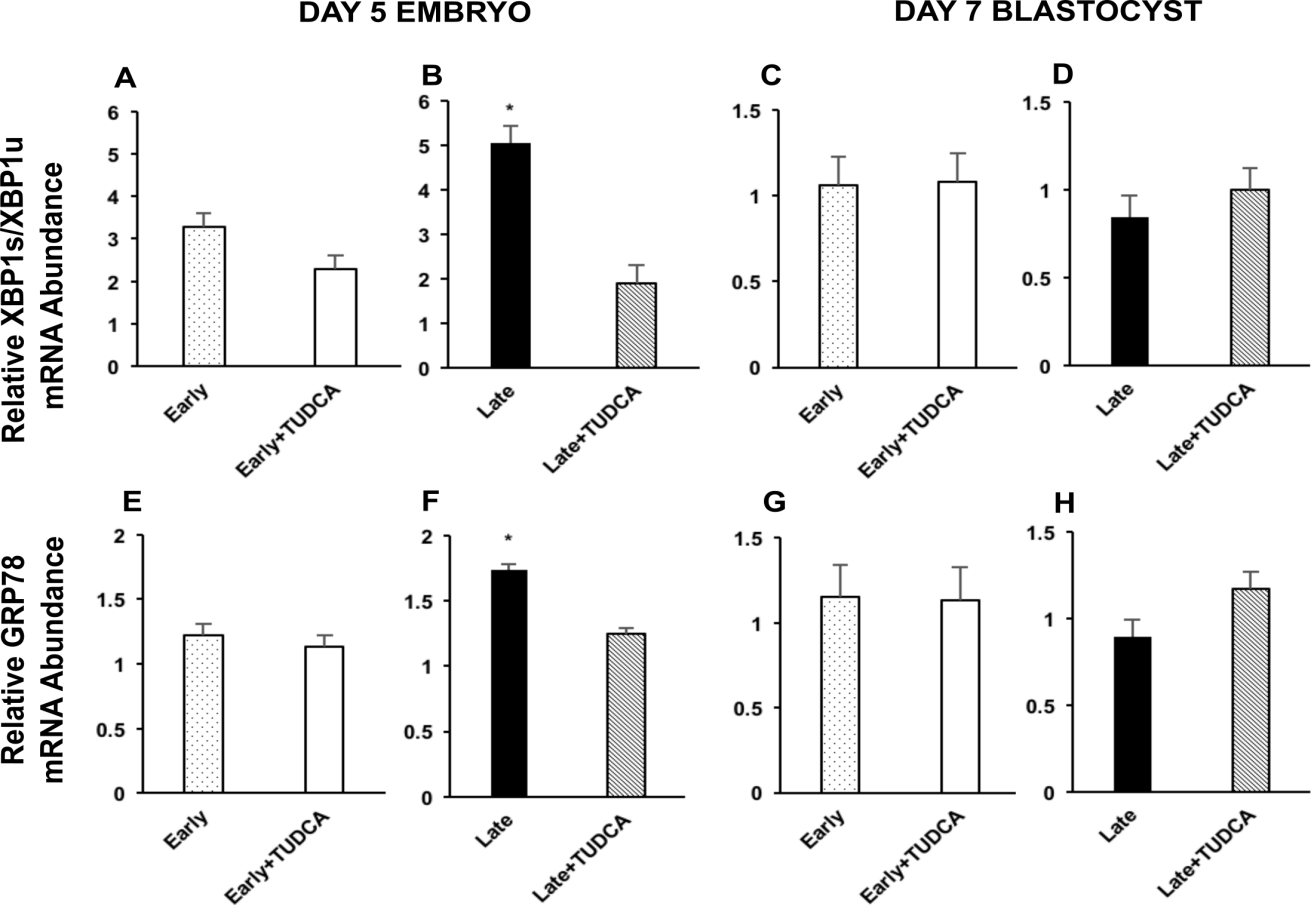
ER stress in the developing embryo and blastocyst: mRNA abundance. Comparison of ER stress marker mRNA abundance in embryos to evaluate ER stress status. XBP1s/XBP1 mRNA abundance in the early-cleaving day 5 embryo, n = 6 (A) and late-cleaving day 5 embryo, n = 6 (B). XBP1s/XBP1 mRNA abundance in the early-cleaving blastocyst, n = 6 (C) and late-cleaving blastocyst, n = 6 (D). GRP78 mRNA abundance in the early-cleaving day 5 embryo, n = 6 (E) and late-cleaving day 5 embryo, n = 6 (F). GRP78 mRNA abundance in the early-cleaving blastocyst, n = 6 (G) and late-cleaving blastocyst, n = 6 (H). Each sample represents a pool of 15 day 5 embryos or 5 blastocysts. Day 3 embryos were not evaluated for mRNA since zygotic genome activation is not complete at this time point. Data was collected from 3 replicates for both developing embryo and blastocyst mRNA evaluation. Late = late-cleaving embryos cultured without TUDCA; Late + TUDCA = late-cleaving embryos cultured with TUDCA; Early = early-cleaving embryos cultured without TUDCA; Early + TUDCA = early-cleaving embryos cultured with TUDCA. Statistically significant differences among groups are indicated by * (p < 0.05).

**Fig 3:**
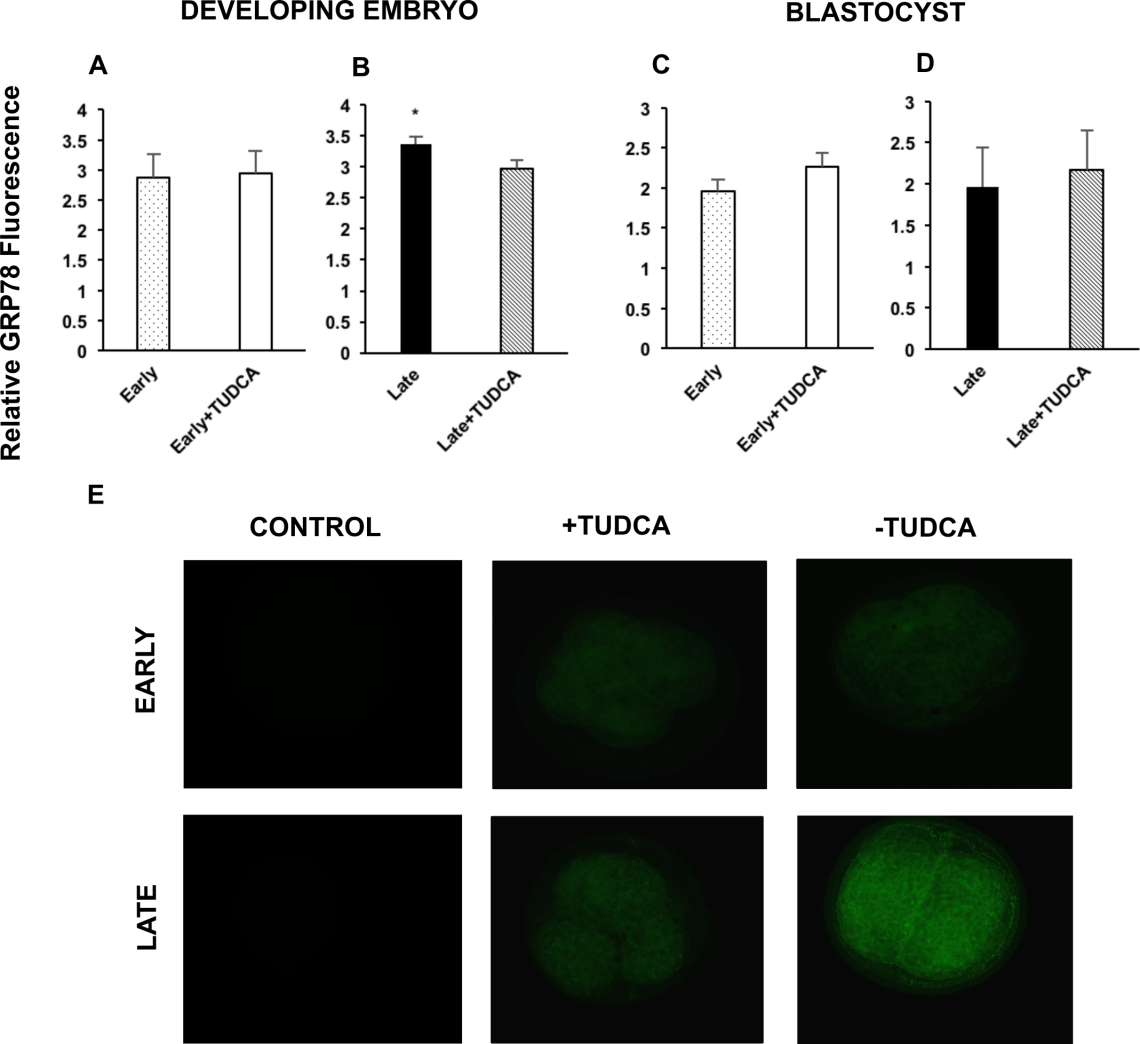
ER stress in the developing embryo and blastocyst: Immunofluorescence. Comparison of ER stress marker immunofluorescence in embryos to evaluate ER stress status. Mean relative GRP78 immunofluorescence in the early-cleaving developing embryo, n = 19 (A) and late-cleaving developing embryo, n = 13 (B). Mean relative GRP78 immunofluorescence in the early-cleaving blastocyst, n = 9 (C) and late-cleaving blastocyst, n = 6 (D). Representative images of GRP78 immunofluorescence for each treatment group in developing embryos (E). Images were captured at 20X magnification. Developing embryos are defined as those collected at day 3 and 5 of culture. Data was collected from 5 and 4 replicates for developing embryo and blastocyst immunofluorescence, respectively. Late = late-cleaving embryos cultured without TUDCA; Late + TUDCA = late-cleaving embryos cultured with TUDCA; Early = early-cleaving embryos cultured without TUDCA; Early + TUDCA = early-cleaving embryos cultured with TUDCA. Statistically significant differences among groups are indicated by * (p < 0.05).

ER stress was evaluated in all embryos by measuring the mRNA abundance of XBP1s and GRP78. Indeed, both were increased in untreated day 5 late-cleaving embryos compared to their untreated counterparts (Fig 2B and 2F, respectively). This effect was not seen in the more developmentally competent day 5 early-cleaving embryos (Fig 2A and 2E, respectively), nor in any embryos of any group that had successfully attained the blastocyst stage (Fig 2C, 2D, 2G and 2H). Furthermore, evaluation of GRP78 protein via immunofluorescence showed a similar trend. Untreated late-cleaving developing embryos had a higher relative GRP78 fluorescence compared to their TUDCA-treated counterparts (Fig 3B and 3E). While treatment with TUDCA caused a significant decrease in GRP78 fluorescence in late-cleaving developing embryos (Fig 3B and 3E), this effect was not seen in more competent early-cleaving developing embryos (Fig 3A and 3E), nor in any embryos of any group that had successfully attained the blastocyst stage (Fig 3C and 3D). Importantly, treatment with TUDCA decreased mRNA abundance of XBP1s in late-cleaving developing embryos to similar levels of early-cleaving developing embryos, treated or not with TUDCA.

**Fig 4:**
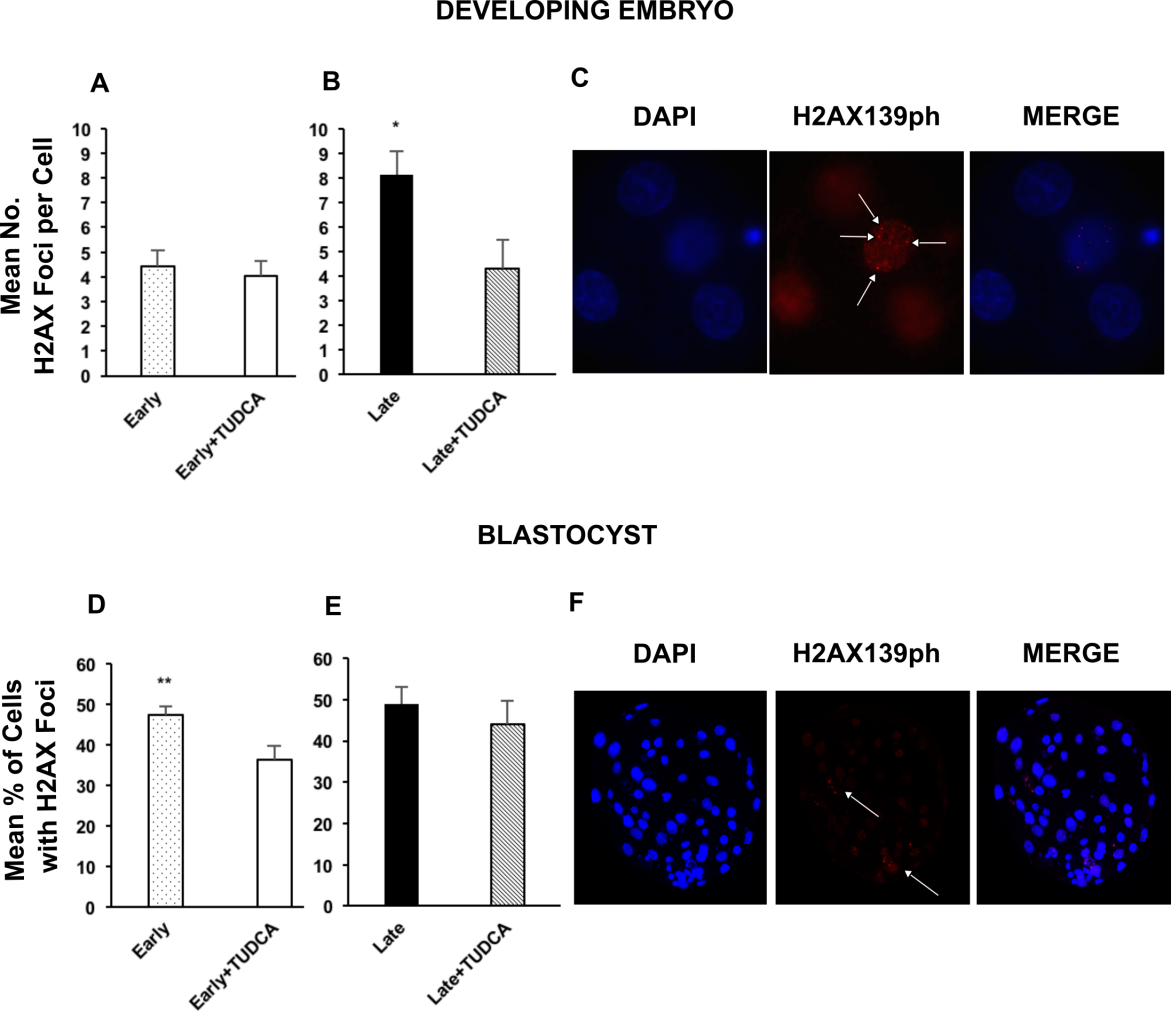
DNA damage in the developing embryo and blastocyst. Evaluation of H2AX139ph foci to assess DNA damage. Comparison of mean H2AX139ph foci per cell in early-cleaving developing embryos, n = 64 (A) and late-cleaving developing embryos, n = 55 (B). Comparison of proportion of cells positive for H2AX139ph foci in early-cleaving blastocysts, n = 52 (D) and late-cleaving blastocysts, n = 23 (E). Representative images of H2AX139ph immunofluorescence in developing embryos (C) and blastocysts (F), indicating nuclei (blue) and the H2AX139ph foci (red) used to assess DNA damage. Images were captured at 20X magnification. Developing embryos are defined as those collected at day 3 and 5 of culture. Late = late-cleaving embryos cultured without TUDCA; Late + TUDCA = late-cleaving embryos cultured with TUDCA; Early = early-cleaving embryos cultured without TUDCA; Early + TUDCA = early-cleaving embryos cultured with TUDCA. Statistically significant differences among groups are indicated by * (p < 0.05) and ** (p < 0.01).

Given DNA damage is an inducer of ER stress, it was also evaluated in both developing embryos and blastocysts. The incidence of DNA double strand breaks was determined by assessing the number of fluorescent foci of H2AX139ph. The mean number of H2AX foci per cell was not different in treated and non-treated early-cleaving developing embryos (Fig 4A). However, inhibition of ER stress by TUDCA treatment decreased the mean number of H2AX foci per cell in late cleaving embryos (Fig 4B). Interestingly, treatment of late-cleaving developing embryos with TUDCA reduced DNA damage to a level similar to that of untreated early-cleaving embryos. At the blastocyst stage, the mean percentage of cells with H2AX foci was decreased by TUDCA treatment in early-cleaving embryos (Fig 4D), but not in late-cleaving embryos (Fig 4E).

## Discussion

Results of this study are based on experiments in parthenogenetically-activated porcine oocytes. This model was selected in place of the standard IVF produced embryos since it was essential to accurately measure the time until the embryo’s first cleavage to carry out the experiments (S1 Table). Furthermore, abnormal oocyte penetration and polyspermy are common problems seen in porcine IVF, which can have detrimental effects on embryo cleavage and development[34]. Finally, parthenogenetic embryo development is an established model for early embryo development[35–39] and the blastocyst rate and quality of embryos produced using this method have been shown to be similar to IVF in pigs[40, 41].

Distinction between early- and late-cleaving embryos is important since the former exhibits improved developmental competence. The findings of the present study demonstrate that ER stress affects embryo cleavage kinetics, and ultimately both the quality and the ability of embryos to develop to the blastocyst stage. Studies in humans and livestock using diverse embryo production methods, including *in vitro* fertilization, intra-cytoplasmic sperm injection, parthenogenetic activation and nuclear transfer, consistently show that late-cleaving embryos have reduced developmental competence[9–12, 16, 34, 42, 43]. Thus, the kinetics of embryo cleavage has been used as a parameter for embryo selection in assisted reproduction in both humans and livestock[35, 36].

The precise causes of compromised development of late-cleaving embryos are not known. These may originate from endogenous sources such as inherent in the gametes, or exogenous sources such as the developmental milieu, of the gametes and embryos. Regardless, late- and early-cleaving embryos exhibit differences in metabolic profile[14], mRNA transcriptome[37–39], mRNA processing[40], and chromatin remodeling[41]. In this study, we found increased UPR activity, an ER stress coping response, as revealed by increased XBP1s and GRP78 mRNA abundance and GRP78 immunofluorescence, in untreated late-cleaving embryos. Remarkably, treatment with TUDCA[44] which has been shown to decrease ER stress in embryos and increase blastocyst rates[29, 30, 33, 45], rescued development and quality of late-cleaving embryos. TUDCA treatment had no apparent negative impacts on early-cleaving embryos. These findings indicate that increased ER stress is a major determinant of reduced developmental potential of late-cleaving embryos, and that embryos at the earliest cell division stages are extremely sensitive to ER stress.

It is known that a variety of common factors are associated with both ER stress and DNA damage[20, 46]. There is also evidence indicating that similar signaling pathways are induced by either DNA damage or ER stress responses in somatic cells[19]. Recent studies showed that late-cleaving embryos have higher incidence of DNA DSBs than early-cleaving embryos[17, 47] and treatment with inhibitors of histone deacetylase enzymes was shown to enhance DNA damage repair[47], as well as reduce ER stress[48] in embryos produced by nuclear transfer. These studies suggest that DNA damage and ER stress may have common effects on embryo cleavage kinetics and development. In our study, higher incidence of DNA damage was detected in untreated late-cleaving developing embryos, as demonstrated by an increase in the mean number of DSBs per cell. These embryos appear to accumulate more DNA damage foci during early development, whereas those that successfully develop to the blastocyst stage are no different from their TUDCA-treated counterparts. Interestingly, early-cleaving blastocysts treated with TUDCA had reduced DNA damage compared to their untreated counterparts, an effect not seen in the late-cleaving blastocysts. This improvement seems to have occurred after genome activation, which coincides with the observation that the DNA damage response is most active near the blastocyst stage[49]. The difference in TUDCA effect at this stage between early and late-cleaving blastocysts is unclear, but may be attributed to greater cellular differentiation, and hence increased activity of DNA damage repair, in the more developmentally advanced early-cleaving groups. Our findings indicate that relief of ER stress had the same positive effect on embryo genome integrity by either decreasing the incidence of DSBs or enhancing genome damage repair[19]. Importantly, our study provides clear evidence linking the genome damage coping response[19] to ER stress coping response pathways in developing embryos.

In conclusion, we show here that increased ER stress and genome damage are present in developing late-cleaving embryos. Pharmacological relief of ER stress also decreased genome damage and improved embryo development. Therefore, reduction of ER stress is a potential means to rescue the developmental competence and quality of late-cleaving and poorly developing embryos. Our findings have significant implications in human and livestock fertility as well as for the refinement of assisted reproductive technologies.

## Materials and methods

### Chemicals and reagents

All chemicals and reagents were purchased from Sigma-Aldrich (St. Louis, MO, USA), unless otherwise specified.

### Animal care

All animal work performed in this study was approved by the Animal Care and Use Committee of McGill in compliance with guidelines from the Canadian Council on Animal Care.

### Oocyte retrieval and maturation

Oocytes were aspirated from ovaries of pre-pubertal gilts collected from a local abattoir (Olymel, S.E.C./L.P, Saint Esprit, Quebec, Canada). Cumulus oocyte complexes (COC) were collected from 3–6 mm follicles and those having at least three layers of cumulus cells and homogeneous cytoplasm were selected for maturation. Groups of 30 COCs were matured for 22 h in 100 μl of maturation medium consisting of TCM 199 (Life technologies, Burlington, ON, Canada), supplemented with 20% of porcine follicular fluid, 1mM dibutyryl cyclic adenosine monophosphate (dbcAMP), 0.1 mg/mL cysteine, 10 ng/mL epidermal growth factor (EGF; Life technologies), 0.91 mM sodium pyruvate, 3.05 mM D-glucose, 0.5 μg/mL LH (SIOUX Biochemical Inc., Sioux Center, IA, United States), 0.5 μg/mL FSH (SIOUX Biochemical Inc.), and 20 μg/mL gentamicin (Life technologies). COCs were transferred to the same IVM medium, but without LH, FSH and dbcAMP, for an additional 20 to 22 h as previously described[17].

### Oocyte activation

After maturation, COCs were denuded with 0.1% hyaluronidase (H3506) to facilitate selection of mature MII oocytes. Mature oocytes were activated for 5 min in 15 μM ionomycin (I0634) followed by 4 h in calcium-free porcine zygote medium (PZM-3) supplemented with 10 mM strontium chloride (255521), 7.5 μg/ml cytochalasin B (C6762) and 10 μg/ml cyclohexamide (C1988), as previously described[50].

### Embryo culture

After activation, embryos were cultured in PZM-3 medium supplemented with 3 mg/ml BSA (A6003). Groups of up 20–30 embryos were cultured in 60 μl droplets under mineral oil at 5% CO_2,_ 95% air and 38.5°C. After 24–30 h, cleavage was assessed and early-cleaving embryos were separated from the rest of the group. After 48–54 h, cleavage was re-assessed and late-cleaving embryos were identified. The categorization of early and late-cleaving embryos according to these time points has been based on previous studies [8, 9, 11, 13, 17, 51]. Any remaining uncleaved embryos were discarded at this stage. Early- and late-cleaving embryos were each divided into two equal groups, one that was subsequently cultured in PZM-3 supplemented with 50 μM TUDCA (Millipore, Billerica, MA, USA, 580549), and another supplemented with an equal volume of vehicle (PBS) (Life Technologies, Carlsbad, CA, USA, 21600–010). The concentration of TUDCA was chosen based on a previous study optimized for porcine embryo culture[30]. Embryos were maintained in culture and collected for analysis at day 3 and 5 (developing embryos), and at day 7 (blastocysts). Embryo quality was evaluated by determining the total cell number of developing and blastocyst stage embryos. qPCR analysis was performed on embryos collected at day 5, and blastocysts collected at day 7. Embryos were not evaluated for mRNA prior to 5 days of culture to ensure that zygotic genome activation had occurred. Embryos cultured beyond 5 days were supplemented with 10% FBS (Life Technologies, 16170–078). All data from the study was collected from a minimum of 3 replicates.

### Evaluation of blastocyst rate and cell number

Blastocyst rates were calculated at day 7 based on the number of cleaved embryos initially maintained in culture. Blastocysts were fixed in 4% formaldehyde (HT501128) for 15 min and permeabilized with 1% Triton X-100 (T8787) in PBS at 37°C for 30 min. Fixed embryos were stained with DAPI (4,6-Diamidino-2-Phenylindole, Dilactate) (Life Technologies, D3571) for 15 min at room temperature, and then mounted on microscope slides in a drop of Mowiol (Polyvinylalcohol, 10852). Cell numbers were counted based on DAPI staining using a Nikon Eclipse 80i microscope (Nikon Instruments Inc., Melville, NY, USA).

### Quantitative real-time PCR

RNA was extracted from embryos using the PicoPure™ RNA Isolation Kit (Life Technologies, KIT0202) and cDNA synthesized using Superscript® VILO™ cDNA Synthesis Kit (Life Technologies, 11754050), according to manufacturer’s recommendations for both procedures. Quantitative real-time PCR was carried out using a CFX Connect™ Real-Time PCR Detection System (Bio-Rad, Hercules, CA, USA, 185–5200) and the iQ™ SYBR® Green Supermix (Bio-Rad, 170–8880). Primers used are listed in Table 1. Thermocycler parameters were 5 min at 95°C, 40 cycles of 15 s at 95°C followed by 30 s at 58°C, and finally 10 s at 95°C and 5 s at 60°C. Samples were run in duplicate and specificity of reaction products were verified by melting-curve analyses. Relative quantities of mRNA were calculated using the ΔΔC_T_ method[52] with the reference 18S ribosomal RNA gene and H2A gene used for normalization. Spliced XBP1 (XBP1s) mRNA abundance was compared to unspliced XBP1 mRNA abundance prior to normalization. All reactions used for quantification had efficiency between 90–110%, R^2^ ≥0.98 and slope values from -3.6 to -3.1.

**Table 1:** Primers used for quantitative real-time PCR.

Gene	Forward Primer	Reverse Primer	Accession No.
**18S**	GACATCTAAGGGCATCACAGA	ACACGGACAGGATTGACAGA	NR_046261.1
**H2A**	GGTGCTGGAGTATCTGACCG	GTTGAGCTCTTCGTCGTTGC	XM_001927727.2
**GRP78**	AGGTGATCTGGTCCTGCTTG	GTCGCTCACCTTCATAGACCTT	XM_001927795.5
**XBP1**	GAGACCAAGGGGAATGGAGC	GCAGAGGTGCACGTAGTCTG	NM_001142836.1
**XBP1s**	CTGAGTCCGCAGCAGGTG	GGCTGGTAAGGAACTGGGTC	NM_001271738.1

### Immunofluorescence analysis

Immunofluorescence experiments were performed as previously described with minor alterations[11, 17]. Embryos were fixed and permeabilized as described above for evaluation of blastocyst cell number. Embryos were stored at 4°C in 1% Triton X-100 in PBS until immunofluorescence analyses could be performed on all replicates simultaneously, to reduce experimental variability in fluorescence staining. Fixed and permeabilized embryos were incubated for 90 min at room temperature in blocking solution, consisting of 3% BSA (Roche, Basel, Switzerland, 10775835001) and 0.2% Tween-20 (P1379) in PBS, and then overnight at 4°C in the presence of the primary antibody. Primary antibodies used were rabbit polyclonal anti-GRP78 (Abcam, Ab191023) and mouse monoclonal anti-phospho-histone H2A.X (Ser139) (Millipore, 05–636) diluted at 1:100 and 1:400 in blocking solution, respectively. Negative control samples were incubated in blocking solution overnight in the absence of primary antibodies. Samples were then rinsed 3 times for 30 min each in blocking solution before incubation in the dark for 50 min in the presence of 1:1000 diluted secondary antibodies, which were either goat polyclonal anti-rabbit Alexa Fluor 488-conjugated IgG antibody (Life Technologies, A-11008) or goat polyclonal anti-mouse Cy3-conjugated IgG antibody (Jackson ImmunoResearch Laboratories Inc., West Grove, PA, USA, 115-165-146). Embryos were then rinsed in blocking solution for 30 min, incubated for 20 min in 10 μg/ml DAPI, and rinsed once more in blocking solution for 30 min before mounting on slides in Mowiol. Slides were analyzed using a Nikon Eclipse 80i microscope (Nikon Instruments Inc.) and images were captured using a Retiga 2000R monochrome digital camera (Qimaging, Surrey, BC, Canada) and the Simple PCI Imaging Software (Compix, Inc., Sewickly, PA, USA). Total GRP78 fluorescence was recorded, adjusted based on embryo cell number, and normalized against negative controls. H2AX139ph foci greater than 0.3 μm^3^, which indicate sites of DNA damage repair and exclude areas of cell cycle regulation and mitosis[53, 54], were counted in individual nuclei and expressed as the average number of positive foci per cell for developing embryos (collected at day 3 and 5), or as the average number of positive cells for blastocysts (collected at day 7). Cell numbers were counted based on DAPI staining for all embryos and blastocysts analyzed.

### Statistical analyses

All statistical analyses were performed using the JMP 10 program (SAS, Cary, NC, USA). Continuous values satisfying a normal distribution were tested using the one-way ANOVA method and means compared using the Student’s t-test. For data sets not normally distributed, continuous values were tested using the Kruskal-Wallis method and means compared using the Wilcoxon test. Significant differences were attributed for P-values < 0.05.

## Supporting information

S1 Table Staging of pig embryos produced by in vitro fertilization (IVF). (DOCX)
