# Supplementary material for: Relief of endoplasmic reticulum stress enhances DNA damage repair and improves development of pre-implantation embryos
Source: PLoS One. 2017 Nov 3;12(11):e0187717. doi: 10.1371/journal.pone.0187717 (PMC5669469; doi:10.1371/journal.pone.0187717)
Supplement: S1 Table — (DOCX) [file pone.0187717.s001.docx]

**S1 Table. Staging of pig embryos produced by in vitro fertilization (IVF)**

| Assigned Group | Cleavage No. (%) | Blastocysts No. (%) |
| --- | --- | --- |
| Early-cleaving | 10/194 (5.2%) | 1/10 (10%) |
| Late-cleaving | 109/194 (56.2%) | 15/109 (13.8%) |
| Total | 119/194 (61.4%) | 16/119 (13.5%) |

Only ~5 % of oocytes (n=194) have cleaved within the same period when >50 % of parthenotes can be qualified as early-cleaving embryos (1). In addition, early- and late-cleaving IVF embryos resulted in similar rates of development to the blastocyst stage, indicating that the two categories for IVF embryos could not be accurately discriminated. The total development of IVF embryos to blastocyst stage was 13.5 %, which most likely was a consequence of higher incidence of polyspermy as observed in our previous studies (2).

**References**

1. Bohrer RC, Coutinho ARS, Duggavathi R, Bordignon V. The Incidence of DNA Double-Strand Breaks Is Higher in Late-Cleaving and Less Developmentally Competent Porcine Embryos. Biology of Reproduction. 2015; 93(3):59, 1–8. DOI 10.1095/biolreprod.115.130542 PMID:26134870.

2. Nascimento AB, Albornoz MS, Che L, Visintin JA, Bordignon V. Synergistic effect of porcine follicular fluid and dibutyryl cyclic adenosine monophosphate on development of parthenogenetically activated oocytes from pre-pubertal gilts. Reprod Domest Anim. 2010; 45:851-9. doi: 10.1111/j.1439-0531.2009.01368.x PMID: 19416484.
